# Genome-Wide Identification and Stage-Dependent Analysis of Odorant-Binding Protein and Chemosensory Protein Genes in the Summer Fruit Tortrix Moth, *Adoxophyes orana* (Lepidoptera: Tortricidae)

**DOI:** 10.3390/insects17101046

**Published:** 2026-10-12

**Authors:** Yu-Han Liu, Xiu-Lin Chen, Xue Chen, Kun Luo, Guang-Wei Li, Bo-Liao Li

**Affiliations:** 1 Key Laboratory for Applied Ecology on Loess Plateau of Shaanxi Higher Education Institutions, College of Life Sciences, Yan’an University, Yan’an 716000, China; 13060498188@163.com (Y.-H.L.); chenxiulin@yau.edu.cn (X.-L.C.); 19890817085@163.com (X.C.); luok@yau.edu.cn (K.L.); liguangwei@yau.edu.cn (G.-W.L.); 2 Shaanxi Key Laboratory of Research and Utilization of Resource Plants on the Loess Plateau, Yan’an University, Yan’an 716000, China

**Keywords:** *Adoxophyes orana*, OBP, CSP, RNA-seq, expression patterns

## Abstract

The summer tortrix fruit moth, *Adoxophyes orana*, is a major fruit trees pest in Asia and Europe, which rolls leaves for food and generate silk nets to avoid prey by natural enemies. As a polyphagous species whose host plants cover more than 30 species, it relies on the olfactory system to receive odor molecules for feeding and mating. However, the gene resources of olfactory genes remain limited. In this study, we conducted genome-guided RNA-seq analyses and identified 31 putative OBP genes (24 classical, 4 minus-C and 3 plus-C OBPs, 4 PBPs, and 2 GOBPs) and 21 putative CSP genes. These putative *OBPs* were located in 8 chromosomes, with 11 in Chr15 and 6 in Chr16. Two *GOBPs* and four *PBPs* are adjacently located in Chr16, and 18 out of 21 CSPs are concentrated in Chr16. Phylogenetic analyses showed that all the putative OBPs were classified into five clusters, with all GOBPs and PBPs exclusively in cluster III, and all four minus-C OBPs in cluster IV. Expression analyses indicated that putative *AoraOBP7/10* were highly expressed throughout larval stages, putative *AoraOBP5/6/8/12/15/21/25* were abundant in first-instar larvae, and 18 of 31 putative OBPs were mainly expressed in adults. In addition to rich *AoraOBPs* in adult antennae, *AoraOBP18/19/20/22* were surprisingly highly expressed in male abdomens, and putative *AoraCSPs* showed complex expression patterns when combining RNA-seq analyses and RT-qPCR analyses. These results provide a gene resource for future functional studies of OBPs and CSPs, which potentially act as targets for pest management.

## Simple Summary

The summer tortrix fruit moth, *Adoxophyes orana*, is a major pest of fruit trees in Asia and Europe, which rolls leaves for food and generates silk nets to avoid being preyed upon by natural enemies. Its host plants cover more than 30 species, especially fruit trees in Rosaceae. It relies on its olfactory system to choose optimal host plants and call mates. However, apart from the functional research of two GOBPs and PBP3, no comparative genome-wide identification olfactory genes and functional research have been published. With RNA-seq data from samples across developmental stages and adult antennae mapped to the genome of *A. orana*, we annotated and identified two small soluble protein families, odorant binding proteins (OBPs) and chemosensory proteins (CSPs), and further analyzed the expression patterns of these samples. These data will provide gene resources for future functional studies of specific OBPs or CSPs and potential targets for pest management.

## 1. Introduction

The remarkable olfactory system acts as a significant contributor to the extraordinary diversity of insects [1]. The olfactory system consists of the peripheral nervous system (PNS) and central nervous system (CNS) [2,3]. In the peripheral part, when odor molecules enter the pores of sensilla on the antennae, two small soluble protein families, odorant-binding proteins (OBPs) and chemosensory proteins (CSPs), bind and transport these hydrophobic odor molecules to odorant receptors (ORs). ORs combine with the odorant receptor co-receptor (Orco) to form a heterotrimer and transduce chemical signals into electrical signals [4]. Odor molecules are then degraded by odorant-degrading enzymes (ODEs) to prevent continuous stimulation [5,6,7,8]. In the central part, electrical signals are first transmitted to projection neurons (PNs), then to local neurons (LNs) and glomeruli in the antennal lobes. Ultimately, the signals reach the protocerebrum, mushroom body (MB), and lateral horn (LH), thereby stimulating the respective behavioral responses [1,2,9]. The nervous interaction pathway of different orders is generally similar in the CNS, but quite divergent in the PNS [2].

OBPs are small, soluble proteins that transport hydrophobic odor molecules from pores on the sensilla of antennae through hydrophilic hemolymph to ORs on the dendritic membrane of odorant neurons (OSNs) [1,4,6]. OBPs were traditionally classified into classic (6 cysteines), minus-C (<6 cysteines), plus-C (>6 cysteines), and atypical-dimer OBPs based on the number of conserved cysteine residues [10,11,12]. Functionally, OBPs have been categorized into pheromone-binding proteins (PBPs), general odorant-binding proteins (GOBPs), and antennal binding proteins (ABPs) [7,10]. However, an increasing number of studies have confirmed that both GOBPs and PBPs receive and recognize sex pheromones [13,14,15,16,17]. In addition to recognizing host plant volatiles (HPVs), many OBPs (including PBPs, GOBPs, and other OBPs) bind sex pheromones [18,19,20]. Several OBPs participate in immunity [21,22].

CSPs are smaller than OBPs, with four conserved cysteines in the pattern of C1-X6-C2-X18-C3-X2-C4 [23,24]. CSPs showed broad functional roles, including recognition of host plant volatiles [25,26,27], insecticide resistance [28,29], behavioral phase change [30], and behavioral activities [31]. For example, the antennae-enriched BminCSP3 in *Bactrocera manix* has a good affinity for citral [25]. CchiCSP1 from *Callosobruchus chinensis* participated in the recognition of mung bean volatiles [26]. Several CSPs in *Nilaparvata lugens* showed binding affinity for imidacloprid and facilitated its detoxification [28]. DcCSP4 from *Diaphorina citri* increased the tolerance of dinotefuran [29]. *MsCSP17* and *MsCSP18* were indispensable for larval development in *Mythimna separata* [30].

The summer fruit tortrix moth, *Adoxophyes orana*, is a multivoltine fruit leafroller pest distributed in temperate Eurasia [32,33,34,35]. When *A. orana* density is low, larvae mainly feed on leaves and young twigs, which does not cause yield losses. However, as density increases, caterpillars attack fruit, causing significant product losses [33,36]. Its host plants cover more than 30 species, including apple, pear, peach, apricot, jujube, mulberry, and tea [37,38]. This means that *A. orana* has a fascinating olfactory system for detecting HPVs and distinguishing chemical signals. However, research on olfactory-related genes of *A. orana* and other Tortricidae subfamily species remains limited, except for the GOBP and PBP genes [24,39].

Here, we performed a genome-wide identification of *OBP* and *CSP* genes in *A. orana*, followed by chromosomal location and phylogenetic analyses. Then, we compared expression profiles across developmental stages and adult tissues using RNA-seq data and validated them by RT-qPCR. These results will enrich the olfactory gene dataset of *A. orana* and provide a theoretical framework for further exploration of its chemosensory biology.

## 2. Materials and Methods

### 2.1. Insect Rearing

The *A. orana* population was originally collected from an apple orchard of Ganquan County, Shaanxi Province, China, in the summer of 2018. The larvae were fed fresh mulberry leaves, and the adults were reared on a 5% sugar solution. The insects were reared in an artificial climate box (BIC 300, Shanghai Boxun Medical Biological Instrument Co., Ltd., Shanghai, China) at (25 ± 1) °C, (60 ± 10)% R.H., and L:D = 15 h:9 h [17].

### 2.2. Identification of OBP and CSP Genes in A. orana

OBP and CSP genes were identified using the conserved domain (PF01395.18 for OBP, and PF03392 for CSP) with hmmscan (v. 3.3) with an E-value cutoff of 1 × 10^−10^. Then, the coding regions were predicted using the open reading frame finder online platform (ORFfinder) (https://www.ncbi.nlm.nih.gov/orffinder/) (accessed on 17 March 2026). Amino acid sequences were analyzed on ExPASy (https://web.expasy.org/cgi-bin/protparam/protparam, accessed on 25 March 2026). Signal peptides of AoraOBPs and AoraCSPs were predicted using SignalP 6.0 [40]. The locations of these genes on chromosomes were visualized on the online platform MapGene2Chrom (http://mg2c.iask.in/mg2c_v2.1/, accessed on 24 March 2026). The conserved motifs of OBP and CSP sequences were predicted using MEME (v. 5.5.3) with a minimum motif width of 10 and maximum motif width of 150 [41].

### 2.3. Phylogenetic Analysis of OBP and CSP Amino Acid Sequences

The predicted open reading frame (ORF) sequences of OBP and CSP family genes from eight other species in Lepidotera were downloaded from the NCBI website, including *Bombyx mori* (NCBI accession number: GCF_030269925.1) [42], *Choristoneura fumiferana* (NCBI accession number: GCF_025370935.1) [43], *Carposina sasakii* (NCBI accession number: GCA_014607495.2) [44], *Grapholita molesta* (NCBI accession number: GCA_022674325.2) [45], *Ostrinia furnacalis* (NCBI accession number: GCF_004193835.3) [46], *Pieris rapae* (NCBI accession number: GCA_905147735.1), *Plutella xylostella* (NCBI accession number: GCF_932276165.1) [47], and *Spodoptera littura* (NCBI accession number: GCF_002706865.2) [48]. After alignment with MUSCLE (v. 3.8.1551) [49], a maximum likelihood phylogeny was constructed using IQ-TREE 2 (v.2.0.7) [50]. Bootstrap support was assessed with 1000 replicates. The built-in ModelFinder tool was used to determine the most appropriate substitution model based on the Bayesian Information Criterion (BIC). Subsequent analyses used the best-fit model: WAG + R8 for OBPs and LG + R8 for CSPs.

### 2.4. RNA-seq-Based Expression Patterns of AoraOBPs

For stage-specific RNA-seq analyses, total RNA of each sample was extracted from eighty eggs, sixty 1st-instar larvae, fifteen 3rd-instar larvae, five 5th-instar larvae, four pupae of each sex on the 6th day after pupation, and three adults of each sex on the third day after eclosion to ensure comparable total RNA amounts. We used three replicates for each stage. We extracted two additional RNA samples from 100 pairs of female and male antennae without replicates. All samples were frozen at −80 °C until RNA extraction. Total RNA was extracted using AG RNAex Pro Reagent (Accurate Biotechnology, Changsha, China). Half of the RNA of each sample was used for RNA-seq, and the remaining for RT-qPCR analyses. Total RNA quantity and purity were assessed using the RNA 1000 Nano Lab Chip Kit (Agilent, Santa Clara, CA, USA). High-throughput RNA sequencing was performed on the Illumina NovaSeq platform in PE150 mode (Majorbio, Shanghai, China).

The raw RNA-seq reads were trimmed using fastp (v. 0.20.1) to remove adapters and low-quality reads [51]. RNA-seq clean reads were mapped to the assembled genome using HiSat2 (v. 2.1.0). Transcriptomes were reconstructed, and the expressions were quantified using StringTie (v. 2.2.1) [52]. Differentially expressed (DE) genes were identified using the edgeR package (v. 4.10.1) with thresholds of |*log*_2_*FC*| > 1 and *FDR* < 0.01 [53]. TPM values for *AoraOBPs* were log2-transformed and normalized (TPM + 1), and cluster analyses were conducted using the pheatmap package (v. 1.0.13).

### 2.5. cDNA Synthesis and RT-qPCR

First-strand cDNA was generated following the instructions of the HiScript III 1st Strand cDNA Synthesis Kit (+gDNA wiper) (Vazyme, Nanjing, China). RT-qPCR amplifications were run on a StepOnePlusTM Real-Time PCR System (ABI, Carlsbad, CA, USA) utilizing ChamQ Blue Universal SYBR qPCR Master Mix (Vazyme, Nanjing, China). Each 20 µL reaction mixture consisted of 10 µL of 2 × ChamQ Blue Universal SYBR qPCR Master Mix, 8.2 µL of nuclease-free water, 0.4 µL of each primer (10 µM), and 1 µL of cDNA. All samples were analyzed using three distinct biological replicates, with each evaluated across three technical replicates. Relative expressions were calculated using the 2^−ΔΔCt^ method, with *EF2* (NCBI accession number: PZ727285) and *SOD2* (NCBI accession number: PZ727286) as the reference genes. *EF2* and *SOD2* ranked the two most stable genes across developmental stages and among sexes. We analyzed and visualized the RT-qPCR data in R (v. 4.6.0). Considering the homogeneity of variance of relative expression among stages or tissues, all data were transformed with the formula log_2_(Relative expression + 1) before parametric tests. Primers are listed in Table S1.

## 3. Results

### 3.1. Identification and Sequence Analysis of AoraOBPs

A total of 31 putative *AoraOBP* genes, each with a complete open reading frame (ORF), were identified from the genome of *A. orana*, including four *PBPs* and two *GOBPs* (Table 1). The lengths ranged from 387 (AoraOBP23) to 552 (AoraOBP20), encoding 128 to 183 amino acids. The molecular weight (MW) of deduced proteins ranged from 14.94 (AoraOBP1) to 20.36 kDa (AoraOBP20). Twenty-six out of 31 AoraOBPs were predicted to possess a putative N-terminal signal peptide, with an average length of 19.7 amino acid residues (Table 1). Twenty-one candidate *AoraCSP* genes were identified from the genome of *A. orana* (Table 2). The lengths of ORFs ranged from 288 (*AoraCSP13*) to 447 (*AoraCSP3*), encoding 95 to 148 amino acid sequences. The molecular weight (MW) of deduced proteins ranged from 10.65 (AoraCSP13) to 17.00 kDa (AoraCSP3). All AoraCSPs were predicted to possess a putative N-terminal signal peptide, with an average length of 17.38 amino acid residues (Table 2).

### 3.2. Chromosomal Locations of OBP and CSP Genes

**Figure 1:**
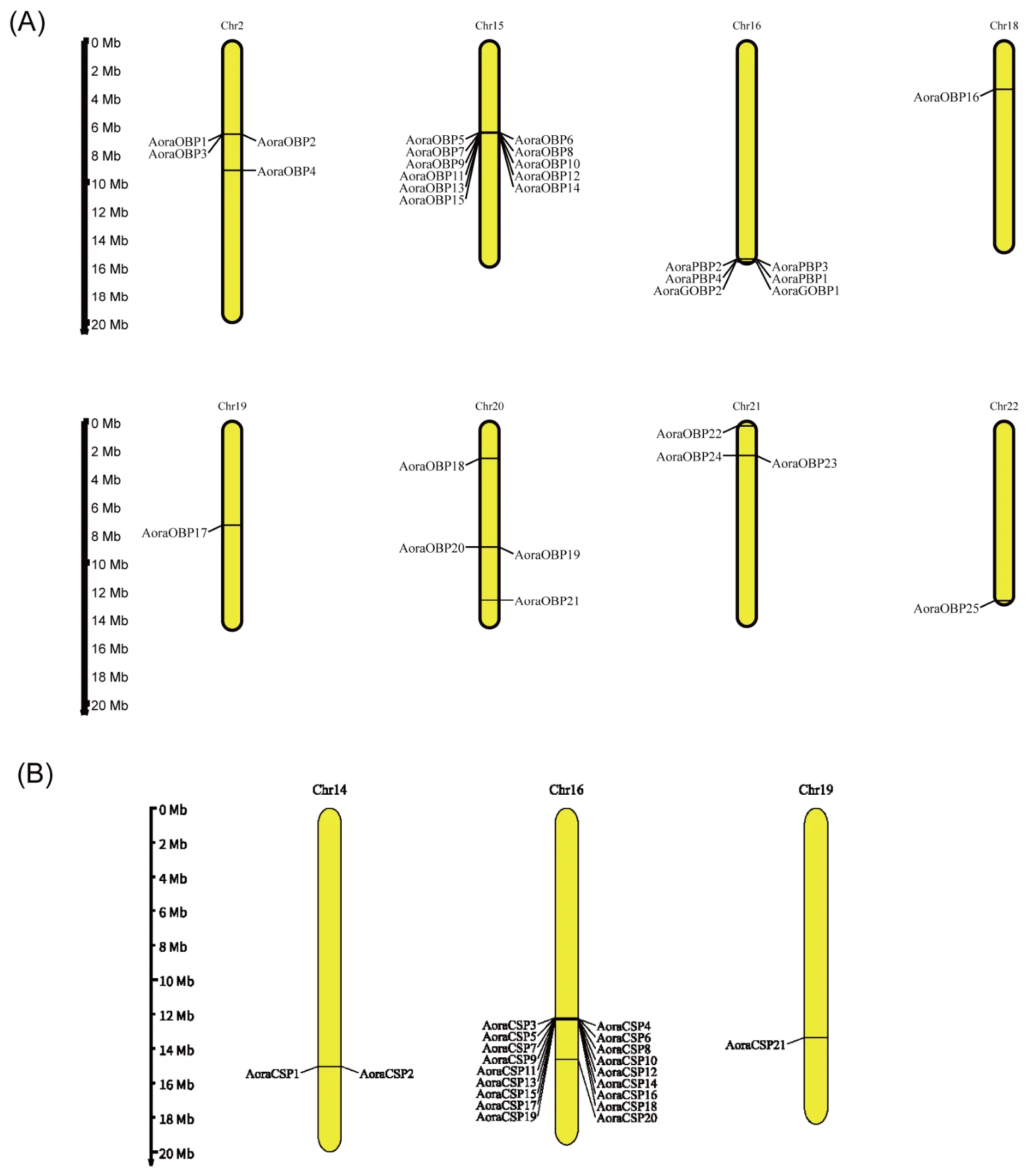
Chromosome distribution of (**A**) OBP and (**B**) CSP family genes in *Adoxophyes orana*.

As shown in Figure 1, as many as 11 putative *AoraOBPs* were located in Chr15 in a cluster. Four, four, and three putative *AoraOBPs* were located in Chr2, Chr20, and Chr21, respectively. Interestingly, all PBP and GOBP genes were found exclusively in Chr16. Eighteen putative *AoraCSPs* were clustered in Chr16, AoraCSP1/2 in Chr14, and only AoraCSP21 in Chr19 (Figure 1).

### 3.3. Protein Motifs and Conserved Domains of AoraOBPs and AoraCSPs

**Figure 2:**
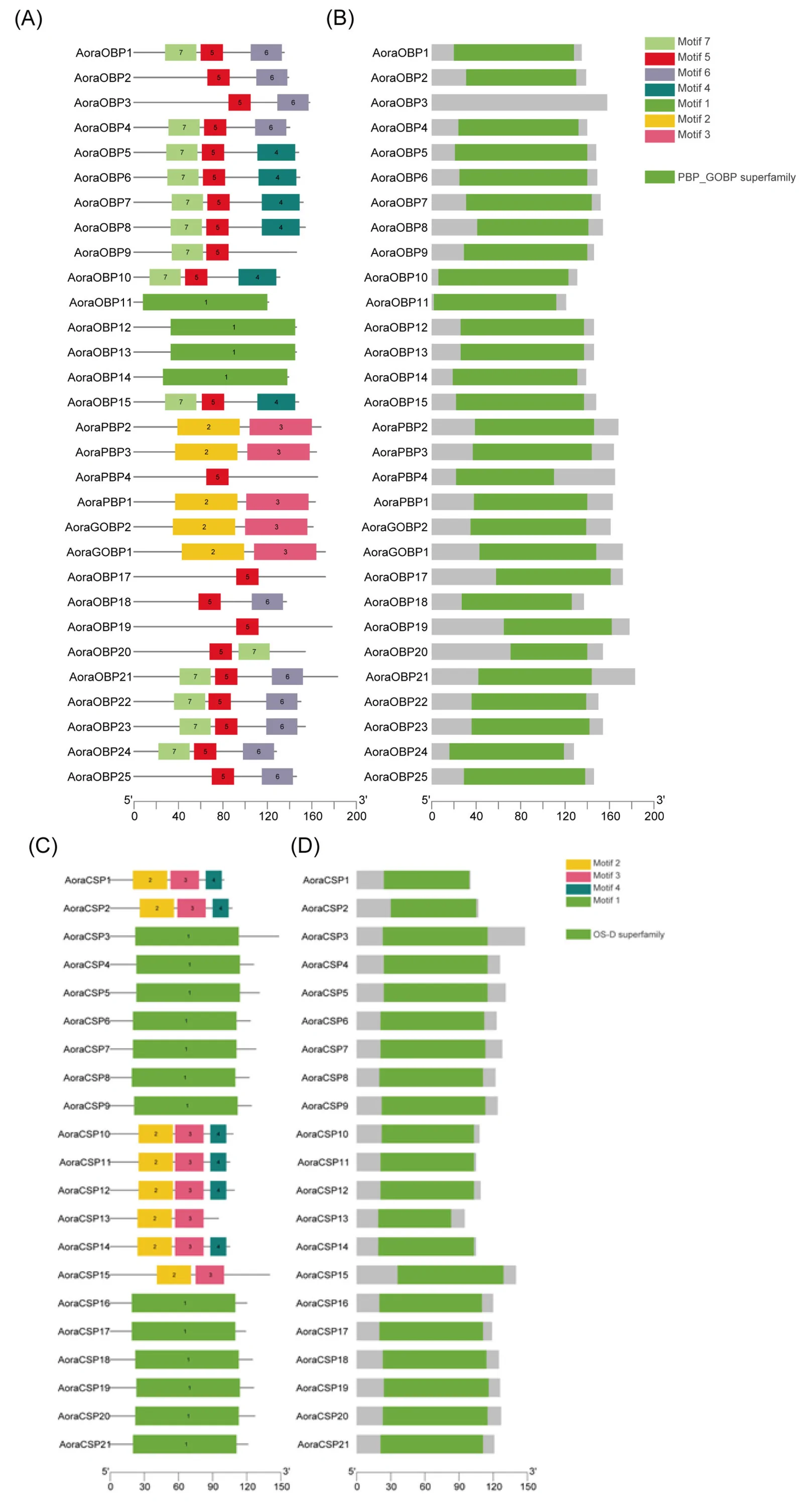
(**A**,**C**) Conserved motifs and (**B**,**D**) domains of OBPs and CSPs of *Adoxophyes orana*.

Motif patterns indicated that AoraOBP11/12/13/14 presented motif1, AoraPBP1/2/3 and AoraGOBP1/2 presented motif 2 and motif 3, and the other AoraOBPs and AoraPBP4 presented at least motif 5 (Figure 2A). Conserved domain analysis indicated that all OBPs contained the PBP_GOBP superfamily domain, except AoraOBP3 (Figure 2B). Thirteen out of 21 putative AoraCSPs presented motif 1, and the other eight owned at least motif 2 and motif 3 (Figure 2C). All putative CSPs contained the OS-D superfamily domain (Figure 2D).

### 3.4. Phylogenetic Analysis of OBPs and CSPs

**Figure 3:**
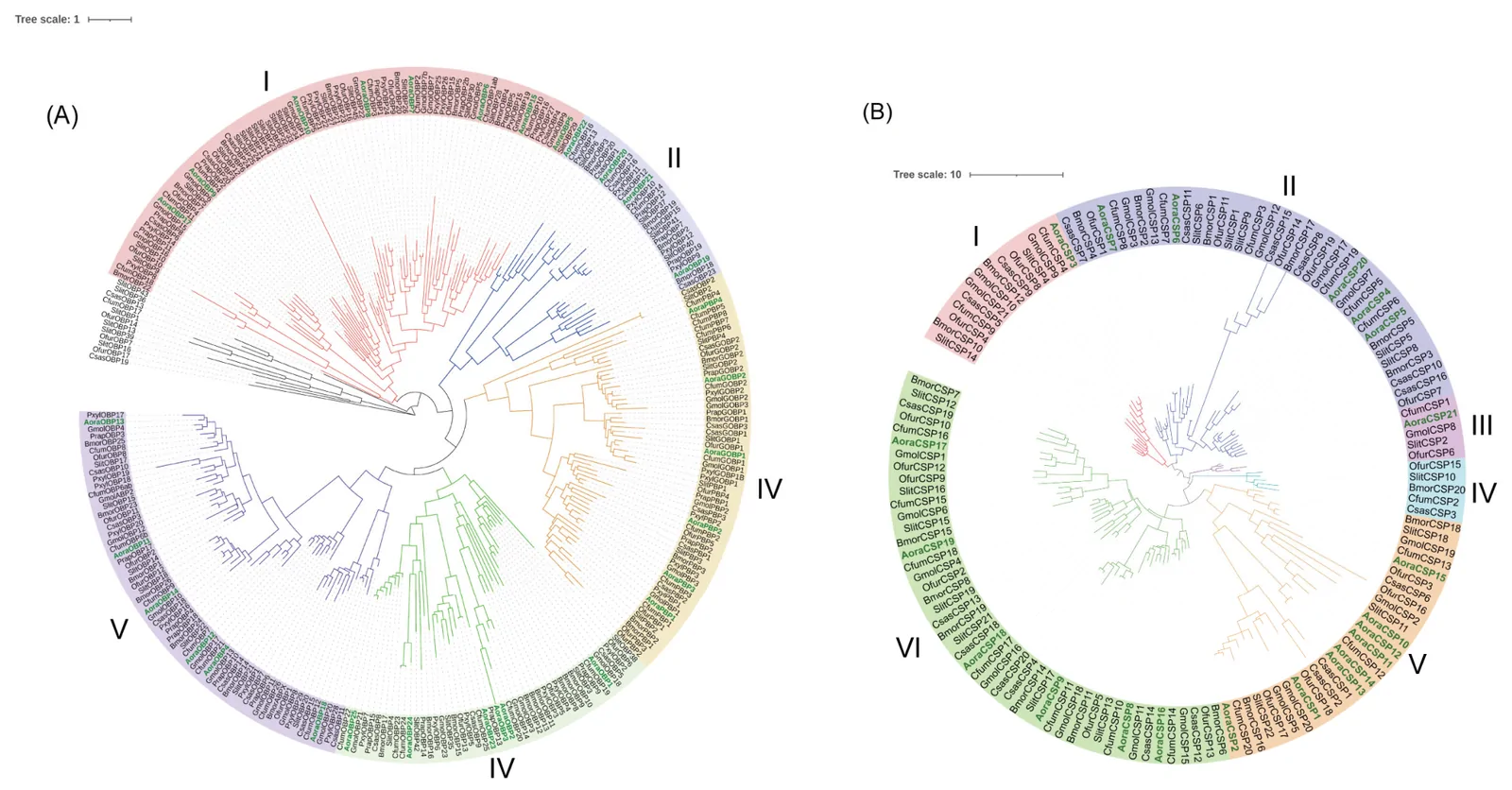
The maximum likelihood phylogenetic tree of putative (**A**) OBP and (**B**) CSP amino acid sequences among *Adoxophyes orana* and other insect species from Lepidoptera. *Aora*, *Adoxophyes orana*. *Bmor*, *Bombyx mori*. *Cfum*, *Choristoneura fumiferana*. *Csas*, *Carposina sasakii*. *Gmol*, *Grapholita molesta*. *Ofur*, *Ostrinia furnacalis*. *Prap*, *Pieris rapae*. *Pxyl*, *Plutella xylostella*. *Slit*, *Spodoptera littura*.

Multiple alignment of OBP amino acid sequences from *A. orana* showed that putative AoraOBP1/2/3/23 belong to minus-C OBP, AoraOBP16/22/24 belong to plus-C OBP, and the other OBPs had six conserved cysteine residues (Table 1). Using the same methodology, we identified candidate OBPs in eight other lepidopteran species: *B. mori*, *C. fumiferana*, *C. sasakii*, *G. molesta*, *O. furnacalis*, *P. rapae*, *P. xylostella*, and *S. littura*. The results indicated that the number of putative OBPs ranged from 24 to 47 among these species (Figure S1). Phylogenetic analysis revealed that the OBP family comprises five subfamilies. Interestingly, all PBP and GOBP amino acid sequences are exclusively clustered in cluster III (Figure 3A). All putative minus-C OBPs (AoraOBP1, AoraOBP2, AoraOBP3, and AoraOBP23), alongside a plus-C OBP (AoraOBP24), belong to cluster IV (Figure 3A).

The number of putative CSPs ranged from 18 to 26 among these species (Figure S1). Phylogenetic analysis indicated that the CSP family consists of six subfamilies. Most putative CSPs from *A. orana* were closely clustered with putative CSPs from *C. fumiferana* (Figure 3B).

### 3.5. Expression Profiles of Putative AoraOBP Genes

**Figure 4:**
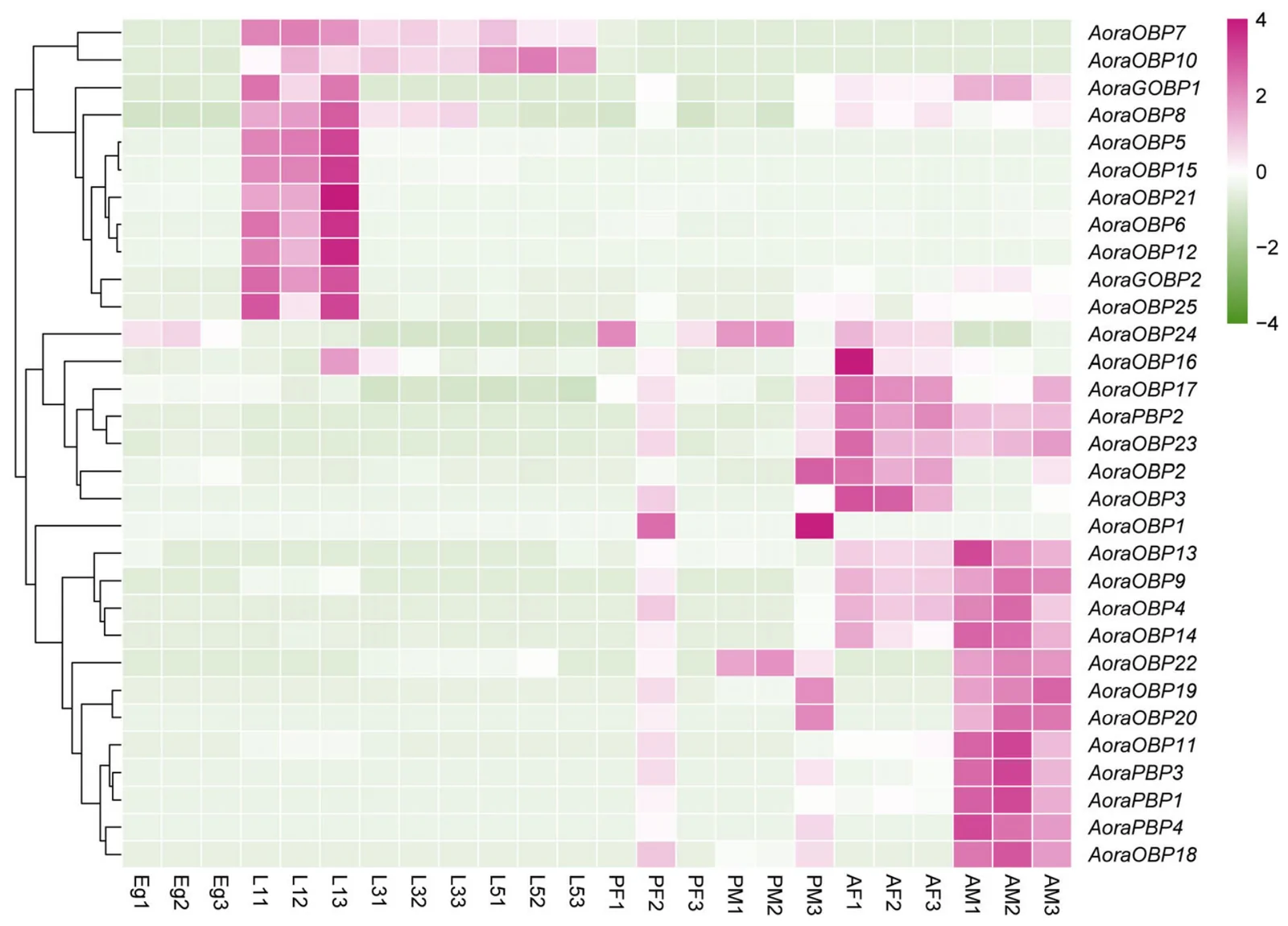
Expression levels of *AoraOBPs* based on RNA-seq TPM data across developmental stages. Eg, L1, L3, L5, PM, PF, AM, and AF represent whole bodies of eggs, 1st-instar larvae, 3rd-instar larvae, 5th-instar larvae, male pupae, female pupae, male adults, and female adults, respectively. The color scale on the right ranges from the lowest (green) to the highest (red) normalized TPM.

The quality control results of RNA-seq data are shown in Table S2. PCA was performed to assess the reproducibility of transcriptomic expression levels among replicates (Figure S2). Cluster analysis of stage-specific RNA-seq data showed that putative *AoraOBP7/10* were highly expressed from the first to the fifth instar. Putative *AoraOBP5/15/21/6/12/25* and *AoraGOBP2* were dominantly expressed in the first-instar larvae. *AoraOBP24* was rich in eggs, pupae, and female adults. All other OBP genes were highly expressed in one or both adult sexes (Figure 4).

**Figure 5:**
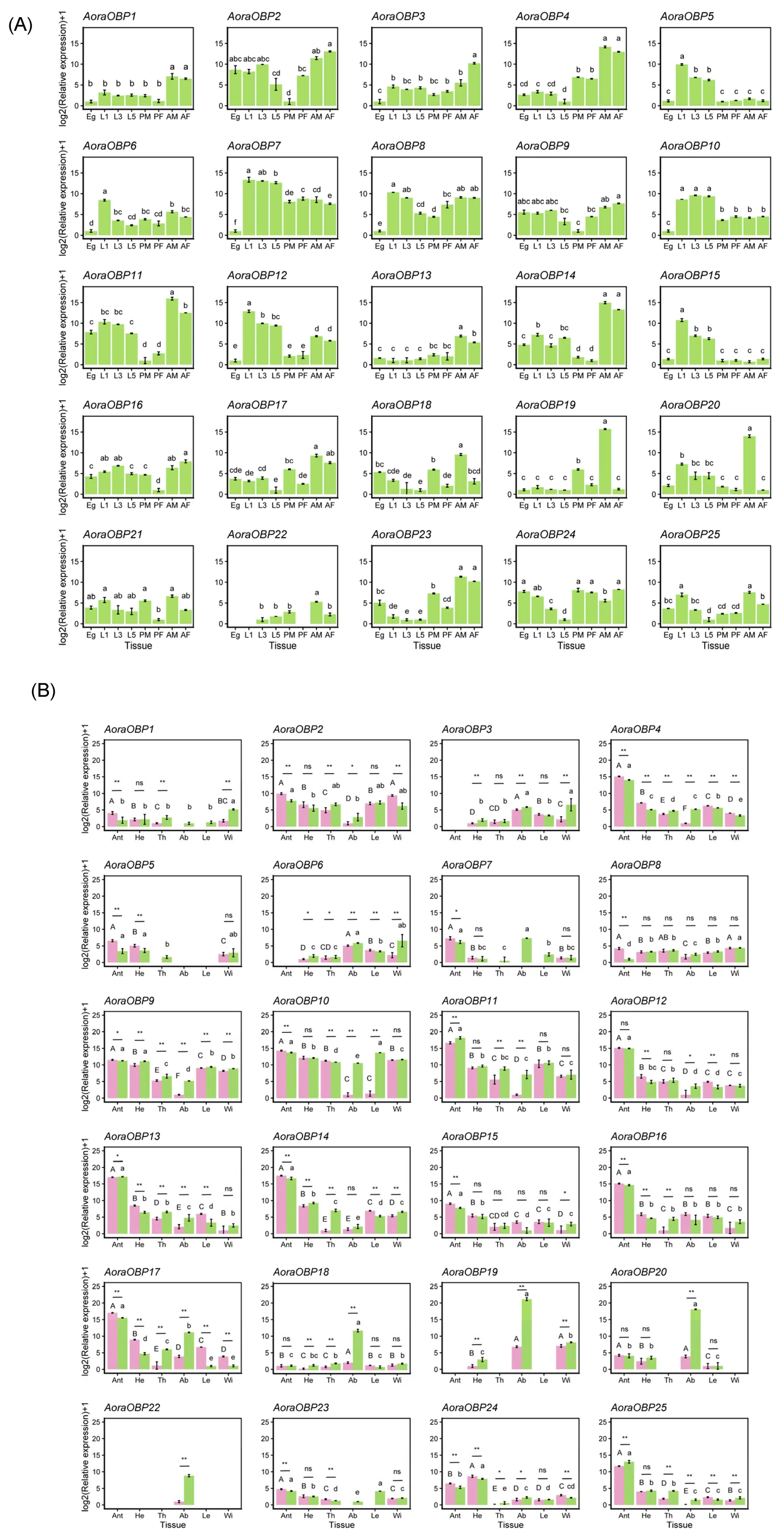
Expression profiles of putative *OBPs* (**A**) across developmental stages and (**B**) among tissues of adult *Adoxophyes orana*. Eg, L1, L3, L5, PM, PF, AM, and AF indicate eggs, 1st-instar larvae, 3rd-instar larvae, 5th-instar larvae, male pupae, female pupae, male adults, and female adults, respectively. Ant, He, Th, Ab, Le, and Wi denote antennae, heads, thoraxes, abdomens, legs, and wings, respectively. Bars are represented as mean ± SD. Pink and light green bars in (**B**) represent female and male moths, respectively. Different uppercase and lowercase letters represent significant differences among tissues from females and males, respectively. * (*p* < 0.05) and ** (*p* < 0.01) represent significant differences between the same tissues of different sexes. “ns” indicate no significant difference between the same tissues of different sexes.

We conducted RT-qPCR to assess the developmental stage, tissue, and sex expression patterns of putative *AoraOBPs*. For most putative *AoraOBPs*, the developmental expression patterns revealed by RT-qPCR were consistent with those derived from RNA-seq data (Figure 5A). *AoraOBP18/19/20*/22 were predominantly expressed in male adults, and *AoraOBP11* was expressed at a significantly higher level in male adults than in female adults. On the contrary, *AoraOBP24* showed significantly higher expression in female adults than in males (Figure 5A). As illustrated in Figure 4 and Figure 5A, *AoraOBP7* and *AoraOBP10* displayed a markedly higher expression level throughout the larval stages. *AoraOBP5/6/8/12* reached its highest expression level in first-instar larvae than in other stages, while *AoraOBP25* showed relatively higher expression in both first-instar larvae and the adults (Figure 5A).

Among adult tissues, putative *AoraOBP4*, *AoraOBP9–17*, and *AoraOBP25* were abundant in adult antennae (Figure 5B). Putative *AoraOBP18/19/20*/22 levels were significantly higher in male abdomens than in the other tissues. Putative *AoraOBP11/17/24/25* were also enriched in the antennae (Figure 5B). Putative *AoraOBP9/24* were abundant in antennae and heads. *AoraOBP3/6/19/22* were not detected in antennae (Figure 5B).

### 3.6. Expression Profiles of Putative AoraCSP Genes

**Figure 6:**
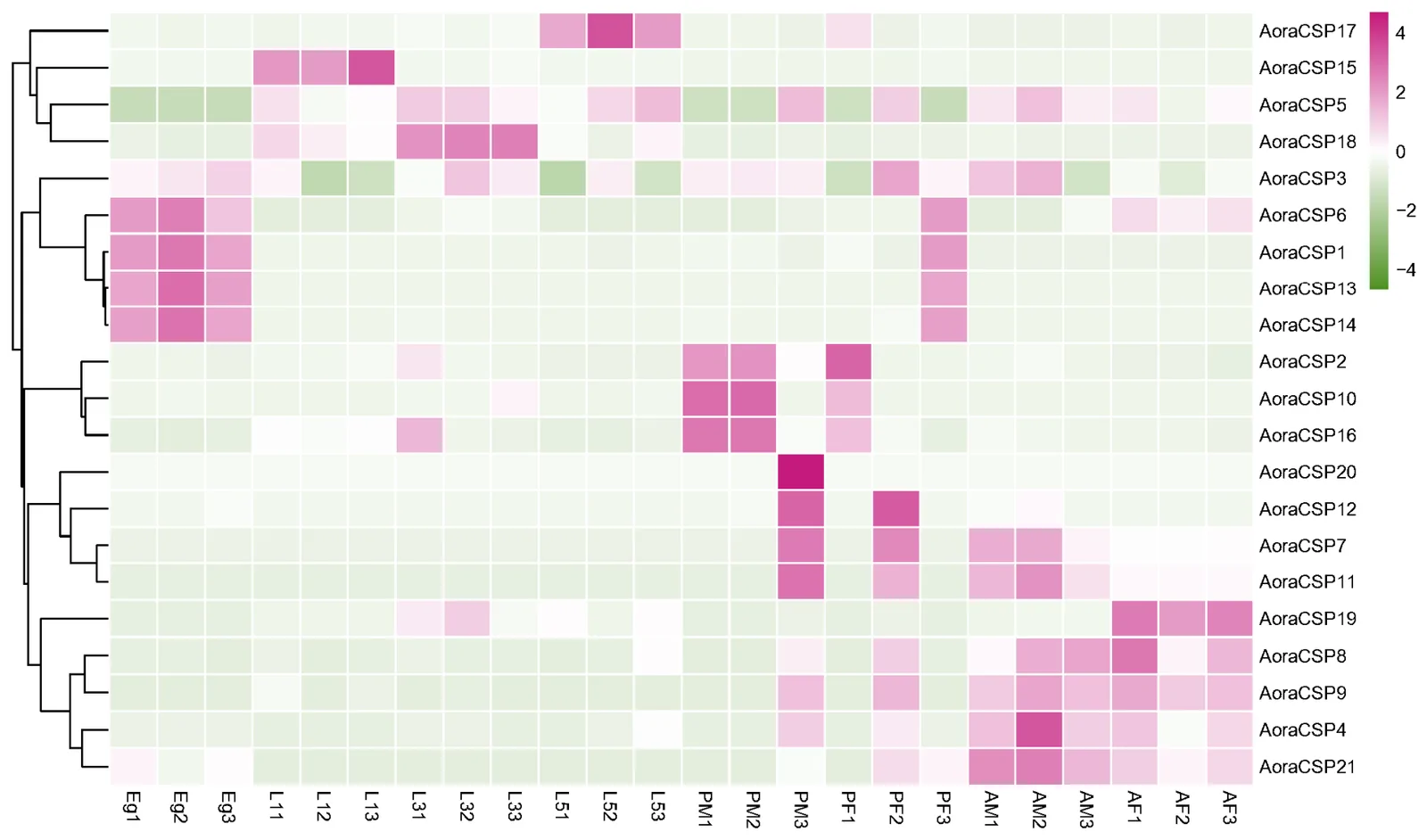
Expression levels of putative *AoraCSPs* based on RNA-seq TPM data across developmental stages. Eg, L1, L3, L5, PM, PF, AM, and AF represent whole bodies of eggs, 1st-instar larvae, 3rd-instar larvae, 5th-instar larvae, male pupae, female pupae, male adults, and female adults, respectively.

Cluster analysis of stage-specific RNA-seq data showed that putative *AoraCSP15* and *AoraCSP17* peaked in the first instar and the fifth instar, respectively. Putative *AoraCSP1/6/13/14* showed predominant expression in eggs, and putative *AoraCSP7/11/19/8/9/4/21* were abundant in the adults (Figure 6). In adults, putative *AoraCSP19* showed female-biased expression in whole-body samples, while the remaining putative *AoraCSPs* showed no significant differences (Figure 6).

**Figure 7:**
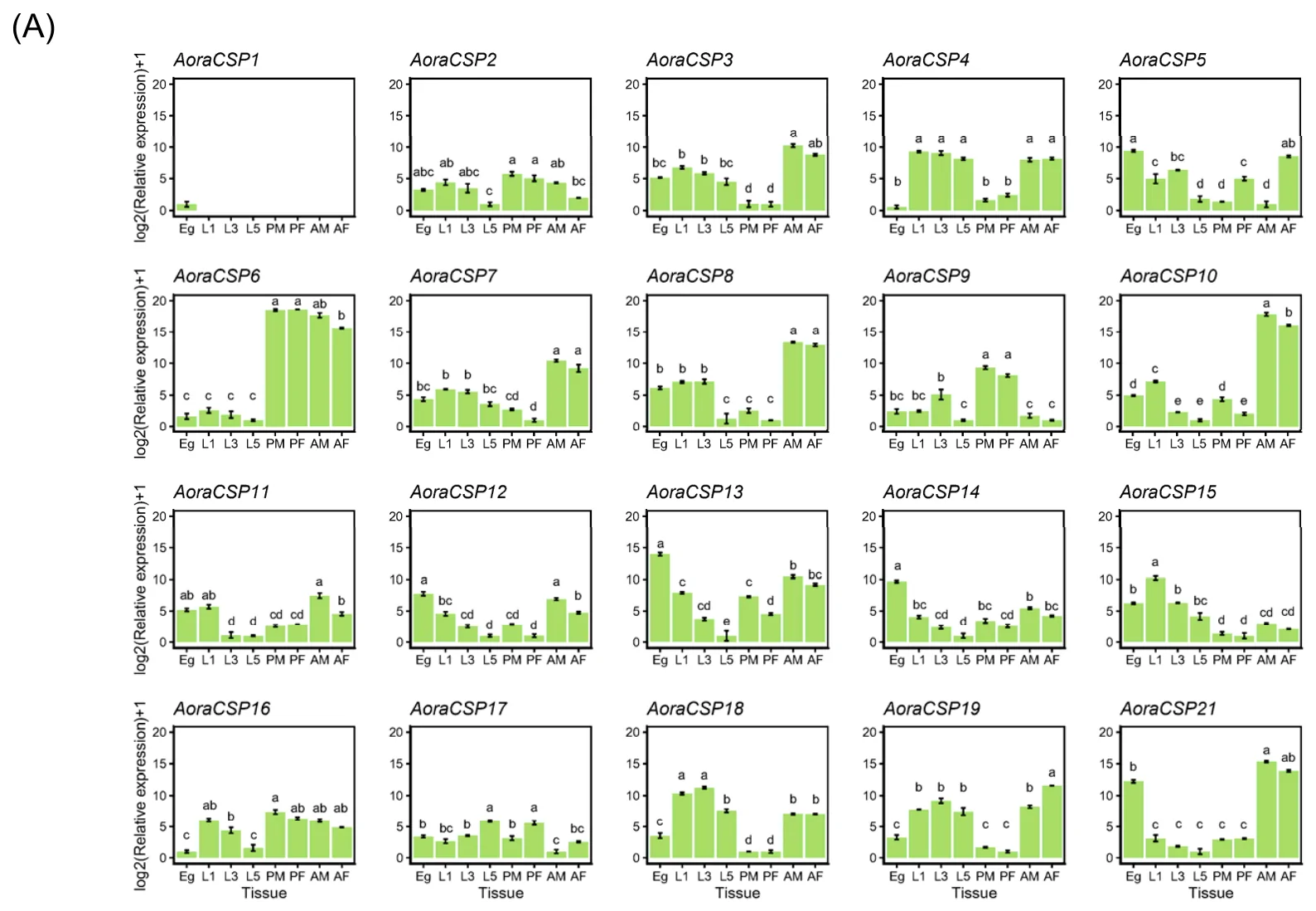

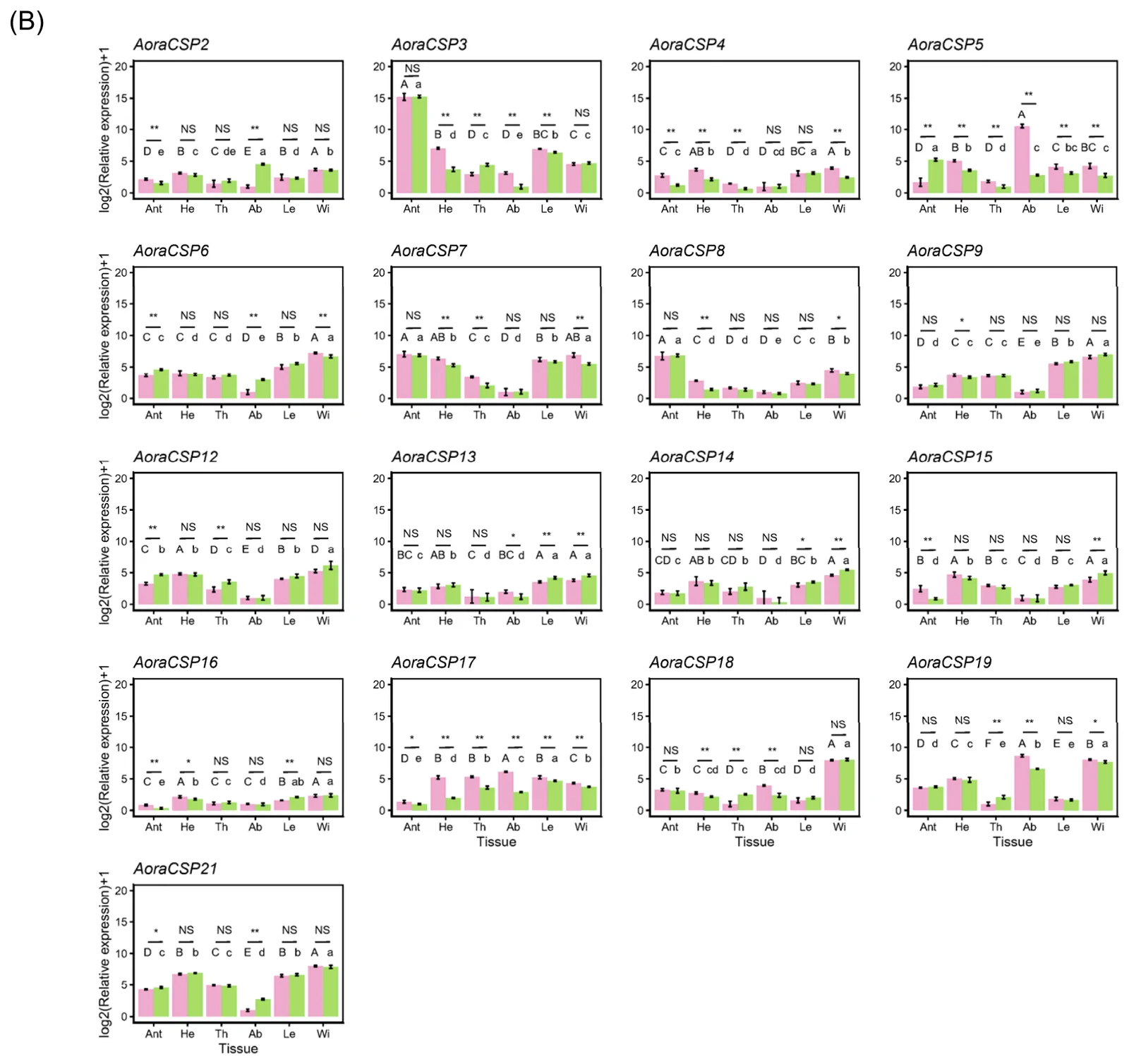
Expression profiles of putative *CSPs* (**A**) across developmental stages and (**B**) among tissues of adult *Adoxophyes orana*. Eg, L1, L3, L5, PM, PF, AM, and AF indicate eggs, 1st-instar larvae, 3rd-instar larvae, 5th-instar larvae, male pupae, female pupae, male adults, and female adults, respectively. Ant, He, Th, Ab, Le, and Wi denote antennae, heads, thoraxes, abdomens, legs, and wings, respectively. Bars are represented as mean ± SD. Pink and light green bars in (**B**) represent female and male moths, respectively. Different uppercase and lowercase letters represent significant differences among tissues from females and males, respectively. * (*p* < 0.05) and ** (*p* < 0.01) represent significant differences between the same tissues of different sexes. “NS” indicate no significant difference between the same tissues of different sexes.

RT-qPCR results showed that putative *CSPs* had more complex developmental stages and tissue expression patterns than *OBPs*. With respect to developmental stages, *AoraCSP1* was detected exclusively in eggs. *AoraCSP5/12/13/14/21* were abundant in both eggs and adults. *AoraCSP3/7/8/10* were rich in adults, and *AoraCSP15* reached its peak in first-instar larvae. *AoraCSP18* and *AoraCSP19* were abundant in both larval and adult stages (Figure 7A).

Among adult tissues, *AoraCSP3* and *AoraCSP8* were enriched in the antennae, while *AoraCSP5* was highly abundant in female abdomens. *AoraCSP17* was significantly higher in all tissues of female adults than in those of males (Figure 7B). *AoraCSP18* was rich in the wings of both sexes. The expression level of *AoraCSP19* was significantly higher in abdomens and wings than in the other tissues (Figure 7B).

## 4. Discussion

The olfactory system is indispensable for insects to locate host plants and mating partners, avoid dangers, and adapt to insecticide stress. OBPs and CSPs transport odor molecules to ORs that are located on the membrane of OSNs. In the last two decades, the widely used RNA-seq and genome sequencing have greatly benefited the identification of OBPs and CSPs. In the present study, we identified 31 putative OBPs and 21 putative CSPs from the genome of *A. orana* (Figure 1). Previously, Jia et al. identified 33 putative OBPs and 21 putative CSPs from the genome of *Spodoptera frugiperda* [23]. A total of 40 putative OBPs were identified from the genome of *Cydia pomonella* [54]. Recently, 47 putative OBPs and 26 putative CSPs were identified from the transcriptome of *Phthorimaea operculella*, whereas 39 putative OBPs and 24 putative CSPs were identified from the transcriptome of *P. absoluta* [55]. A total of 52 putative OBPs and 18 putative CSPs were identified from various tissues of adult *Leguminivora glycinivorella* [56]. Taken together with *B. mori* [42], *C. sasakii* [44,56], *C. fumiferana* [43], *G. molesta* [45,57], *O. furnacalis* [46,58], *P. rapae* [59], *P. xylostella* [47], and *S. litura* [60,61], it was concluded that the number of OBPs fluctuated among species in Lepidoptera, while the number of CSPs remained more stable, approximately around 20, in most Lepidoptera species. However, it could not be excluded that this antenna transcriptome-based annotation usually undercounts gene families.

AoraOBPs with six conserved cysteines belonged to classical OBPs, whereas AoraOBPs lacking C2 and/or C5 belonged to minus-C OBPs, and those with over six cysteines were classified as plus-C OBPs. Within 31 identified putative OBPs in *A. orana*, 24 belonged to classical OBPs, 4 to minus-C OBPs, and 3 to plus-C OBPs. A total of 30 classical, 4 minus-C, 3 plus-C, and 2 atypical OBPs were identified in *C. pomonella* [54]. Two minus-C, three plus-C, and zero atypical OBPs out of 33 were found in *S. frugiperda* [23]. Three minus-C, four plus-C, and zero atypical out of 38 OBPs were identified in *S. litura* [61]. On the contrary, four conserved cysteines were recognized in all putative CSPs from *A. orana* and the other lepidoptera species [23,59,60].

Phylogenetic analyses showed that OBPs in Lepidoptera were classified into five clusters. Interestingly, all PBPs and GOBPs were located in cluster III (Figure 3), suggesting that PBP/GOBP sequences evolved on an independent branch. Chromosomal location indicated that *PBPs* and *GOBPs* of *A. orana* were exclusively and adjacently located in Chr 16 (Figure 1), corresponding to several other lepidopteran insects. Previous studies confirmed that *PBPs* and *GOBPs* were located in Chr 19 of *B. mori* and Chr 8 of *C. pomonella* [53]. Contrary to the chromosomal location of OBPs, the chromosomal locations of CSPs were concentrated. Surprisingly, 18 out of 21 *CSPs* were adjacently distributed in Chr 16 of *A. orana* (Figure 1). Similarly, 20 out of 22 *CSPs* were located in Chr 8 of *S. frugiperda*, likely indicating that the origin of CSP genes comes from gene duplication [23].

RNA-seq analyses revealed that putative *AoraOBP7/10* were rich throughout the larval stage, and putative *AoraOBP5/6/12/15/21/25* were abundant in first-instar larvae (Figure 4). In addition, RT-qPCR results showed that the expression levels of putative *AoraOBP5/12/15* were high in third- and fifth-instar larvae, although lower than first-instar larvae (Figure 5A). Two reasons might explain why more *OBPs* were abundant in newly hatched larvae. First, newly hatched larvae rely on olfactory systems to choose host plants. When *A. orana* start to feed, they will generate silk that adheres to leaves and wrap themselves to hide, and will not relocate feeding places until the leaves are eaten up. Therefore, they do not need so many olfactory proteins to recognize odors in the late-instar larvae until pupation. Second, the weight and size ratio of the head to the whole body decreases as the larval instar increases. In the adults, these genes are all highly expressed in the antennae (Figure 5). In *S. frugiperda*, several *OBPs* were also expressed at higher levels in the earlier instars than the older larvae [23]. Although most *OBPs* were mainly expressed in the adult antennae, some other *OBPs* were rich in the other tissues (Figure 5B). *AoraOBP18/19/20/22* were highly expressed in male abdomens (Figure 5B). We will determine later whether these four genes participate in mating or other functions. Finally, a small part of genes showed different expression patterns calculated between RNA-seq data and RT-qPCR, such as *AoraOBP21*. RNA-seq quantifies fragmented cDNA reads mapped to a gene or transcript, and typically normalized using TPM or FPKM. RT-qPCR reflects the transcripts containing the amplified region, and its results were normalized to the housekeeping gene. As with the exponential phase of qPCR, the fluorescence signal increases exponentially with each cycle, and RT-qPCR is typically more sensitive to fold-change detection for several specific genes. This may explain why the difference in the expression of some genes was detected in RT-qPCR, but not in RNA-seq.

Putative *AoraCSPs* showed more complicated expression profiles across developmental stages. *AoraCSP1/6/13/14* were abundant in eggs, and *AoraCSP15* and *AoraCSP17* were rich in first- and fifth-instar larvae, respectively. *AoraCSP4/7/8/9/11/19/21* were mainly expressed in the adults (Figure 6). In *C. sasakii*, *CsasCSP1* and *CsasCSP12* were surprisingly high in male abdomens, and *CsasCSP5* was abundant in antennae and male wings [62]. *MsepCSP17* was abundant in the larval stage, while *MsepCSP18* was highly expressed in the adults of *Mythimna separata* [30]. The different expression patterns of CSPs suggested divergent functions. CSPs in insects recognize pheromones [63], host plant volatiles [64,65], and insecticides [28,66,67], as well as other functions including development [30], immunity [68], and even acting as effectors that interact with plant defense [69]. Future work will explore tissue expression patterns of *AoraCSPs* in larval and adult stages and their specific functions.

## 5. Conclusions

In summary, we identified 31 putative OBP and 21 CSP family members in the genome of *Adoxophyes orana*. Most putative *OBPs* were located on Chr15 and Chr16, and most putative *CSPs* were located adjacently on Chr16. More than half of *OBPs* were abundant in adult antennae, while *CSPs* showed more complicated expression patterns across developmental stages and among adult tissues. These findings provide the prerequisite data for functional research on this fruit tree pest.

## Figures and Tables

**Table 1:** Characteristics of OBP genes in the genome of *Adoxophyes orana*.

Gene Name	Chr	Direction	Start	End	aa	MW (kDa)	pI	Instability Index	Aliphatic Index	GRAVY	SP	Cys Number	Exon Number	ORF (bp)
AoraOBP1	Chr2	+	6,936,449	6,937,756	135	14.94	9.35	10.05	81.70	−0.346	1–16	4	2	408
AoraOBP2	Chr2	+	6,939,168	6,940,974	139	15.57	8.92	31.88	94.03	0.001	1–18	4	2	420
AoraOBP3	Chr2	+	6,943,630	6,944,591	158	17.58	7.61	31.71	87.59	0.040	No	5	2	477
AoraOBP4	Chr2	+	9,678,895	9,682,688	140	16.16	4.61	35.98	70.29	−0.316	1–17	6	5	423
AoraOBP5	Chr15	−	6,794,564	6,797,510	148	16.55	8.05	25.61	74.53	−0.155	1–20	6	4	447
AoraOBP6	Chr15	−	6,799,464	6,804,055	149	17.02	4.86	36.65	72.08	−0.299	1–21	6	4	450
AoraOBP7	Chr15	+	6,806,060	6,808,003	152	16.88	5.04	50.86	80.26	−0.147	1–22	6	4	459
AoraOBP8	Chr15	−	6,813,530	6,817,329	154	16.67	5.82	38.59	94.35	0.136	1–21	6	4	465
AoraOBP9	Chr15	−	6,823,900	6,826,267	146	16.37	5.54	41.12	77.47	−0.305	1–22	6	4	441
AoraOBP10	Chr15	−	6,831,710	6,833,008	152	16.55	5.29	47.24	79.67	−0.155	1–21	6	4	459
AoraOBP11	Chr15	−	6,839,562	6,843,309	142	16.39	8.44	28.55	72.75	−0.328	1~21	6	5	429
AoraOBP12	Chr15	−	6,843,955	6,847,097	146	16.67	8.57	47.44	88.70	−0.04	1–25	6	5	441
AoraOBP13	Chr15	−	6,854,828	6,857,788	146	17.09	8.57	36.86	58.08	−0.271	1–25	6	4	441
AoraOBP14	Chr15	+	6,859,758	6,862,368	139	15.94	8.42	49.20	49.20	−0.074	1–18	6	5	420
AoraOBP15	Chr15	+	6,864,175	6,866,559	148	16.69	6.08	36.91	62.03	−0.319	1–19	6	4	447
AoraPBP2	Chr16	+	16,225,564	16,227,227	168	19.07	5.37	19.07	72.68	−0.248	1–24	6	3	507
AoraPBP3	Chr16	−	16,231,718	16,233,003	164	18.46	4.96	18.36	102.32	−0.009	1–22	6	3	495
AoraPBP4	Chr16	+	16,234,274	16,236,124	165	17.91	7.61	31.00	91.82	0.158	1–19	6	3	498
AoraPBP1	Chr16	−	16,240,156	16,241,069	163	18.28	5.46	19.62	92.64	−0.093	1–19	6	3	492
AoraGOBP2	Chr16	−	16,244,421	16,246,282	161	18.15	5.19	50.83	84.16	−0.024	1–20	6	3	486
AoraGOBP1	Chr16	−	16,445,221	16,447,303	163	18.62	5.21	42.77	74.29	−0.156	1~19	6	3	492
AoraOBP16	Chr18	+	3,578,148	3,606,158	172	19.07	4.56	45.28	75.00	−0.248	No	7	5	519
AoraOBP17	Chr19	−	7,766,588	7,767,611	137	15.70	5.05	18.95	88.25	−0.043	1–16	6	5	414
AoraOBP18	Chr20	−	2,798,751	2,799,970	178	20.30	9.05	50.13	79.38	−0.662	No	6	3	537
AoraOBP19	Chr20	+	9,344,907	9,345,364	154	17.68	7.59	35.62	93.05	−0.173	1–18	6	1	465
AoraOBP20	Chr20	+	9,368,144	9,376,220	183	20.36	8.75	51.57	93.72	−0.031	1–17	6	4	552
AoraOBP21	Chr20	+	16,379,315	13,385,202	150	17.18	8.93	42.13	81.93	−0.291	1–21	6	4	453
AoraOBP22	Chr21	+	352,404	354,221	154	16.88	8.40	36.17	82.40	−0.086	1–23	8	4	465
AoraOBP23	Chr21	−	2,575,488	2,579,594	128	15.02	9.43	47.76	90.62	−0.349	No	4	3	387
AoraOBP24	Chr21	−	2,583,522	2,586,313	146	16.78	9.29	35.66	73.49	−0.664	No	7	4	441
AoraOBP25	Chr22	−	13,405,161	13,405,571	136	14.56	4.51	26.35	86.99	0.095	1–18	6	1	411

**Table 2:** Characteristics of putative CSP genes in the genome of *A. orana*.

Gene Name	Chr	Direction	Start	End	aa	MW (kDa)	pI	Instability Index	Aliphatic Index	GRAVY	SP	Cys Number	Exon Number	ORF (bp)
AoraCSP1	Chr14	+	12,757,505	12,763,703	100	11.22	10.14	56.84	95.80	−0.190	15	4	3	303
AoraCSP2	Chr14	+	12,764,312	12,775,076	107	11.88	9.36	45.20	93.83	−0.145	18	4	3	324
AoraCSP3	Chr16	+	10,372,573	10,375,643	148	17.00	5.25	39.87	71.76	−0.714	18	4	3	447
AoraCSP4	Chr16	+	10,383,218	10,431,527	126	14.49	8.50	44.90	89.76	−0.458	18	4	3	381
AoraCSP5	Chr16	−	10,395,154	10,397,608	131	14.76	9.32	42.15	95.95	−0.240	18	4	2	396
AoraCSP6	Chr16	−	10,398,277	10,402,832	123	14.28	8.23	31.39	72.11	−0.624	18	4	2	372
AoraCSP7	Chr16	+	10,403,582	10,407,005	128	14.53	5.94	27.97	95.31	−0.245	17	4	3	387
AoraCSP8	Chr16	−	10,431,855	10,433,570	122	14.06	8.30	36.40	82.30	−0.411	17	4	3	369
AoraCSP9	Chr16	−	10,436,684	10,440,838	124	14.22	9.28	29.47	87.18	−0.434	17	4	3	375
AoraCSP10	Chr16	−	10,442,890	10,444,731	108	12.18	6.30	23.64	86.76	0.007	19	4	3	327
AoraCSP11	Chr16	−	10,445,586	10,448,569	105	11.71	6.17	51.91	80.00	−0.014	19	4	3	318
AoraCSP12	Chr16	−	10,451,008	10,453,276	109	12.53	7.52	33.47	66.24	−0.168	19	4	3	330
AoraCSP13	Chr16	−	10,457,383	10,459,404	95	10.65	5.25	40.79	100.74	0.102	18	4	3	288
AoraCSP14	Chr16	+	10,469,763	10,471,012	105	12.27	6.89	34.34	90.19	−0.297	18	4	3	318
AoraCSP15	Chr16	+	10,479,745	10,483,553	140	15.82	5.41	34.98	81.64	−0.255	18	4	3	423
AoraCSP16	Chr16	+	10,485,487	10,487,457	120	13.82	8.88	29.81	76.33	−0.535	16	4	3	363
AoraCSP17	Chr16	−	10,492,987	10,498,886	119	13.44	5.47	33.59	80.42	−0.307	16	4	3	360
AoraCSP18	Chr16	−	10,501,261	10,503,947	125	13.90	6.57	41.45	94.48	−0.194	16	4	3	378
AoraCSP19	Chr16	+	10,509,751	10,511,389	126	14.21	6.30	31.93	82.06	−0.383	16	4	3	381
AoraCSP20	Chr16	+	12,441,799	12,445,548	127	14.80	8.60	32.90	89.13	−0.424	18	4	3	384
AoraCSP21	Chr19	+	11,330,975	11,333,660	121	13.73	8.92	34.06	87.11	−0.507	16	4	2	366

## Data Availability

The raw data of *A. orana* transcriptomes were deposited in https://ngdc.cncb.ac.cn with the GSA run accession number from CRR2977930 to CRR2977955.

## Supplementary Materials

The following supporting information can be downloaded at https://www.mdpi.com/article/10.3390/insects17101046/s1, Table S1: Primers for RT-qPCR of *AoraOBPs*, *AoraCSPs*, *AoraEF2*, and *AoraSOD2*; Table S2: Quality control of RNA-seq data from various developmental stages of *A. orana*; Figure S1: Gene number counts and phylogenetic analysis of OBPs across nine lepidopteran species; Figure S2: PCA analysis of RNA-seq data across various developmental stages of *A. orana*.

## Abbreviations

The following abbreviations are used in this manuscript:

PNS	Peripheral nervous system
CNS	Central nervous system
OBP	Odorant binding protein
CSP	Chemosensory protein
OR	Odorant receptor
Orco	Odorant receptor co-receptor
ODE	Odorant-degrading enzyme
PN	Projection neuron
LN	Local neuron
MB	Mushroom body
LH	Lateral horn
GOBP	General odorant binding protein
PBP	Pheromone binding protein
ABP	Antennal binding protein
HPV	Host plant volatile
RT-qPCR	Real-time quantitative polymerase chain reaction
ORF	Open reading frame
