# Supplementary material for: Genome-Wide Identification and Stage-Dependent Analysis of Odorant-Binding Protein and Chemosensory Protein Genes in the Summer Fruit Tortrix Moth, Adoxophyes orana (Lepidoptera: Tortricidae)
Source: Insects. 2026 Oct 12;17(10):1046. doi: 10.3390/insects17101046 (PMC13705382; doi:10.3390/insects17101046)
Supplement: Supplementary file 1 [file insects-17-01046-s001.zip › insects-4555428-supplementary.pdf]

Table S1. Primers for RT-qPCR of *AoraOBPs, AoraCSPs, AoraEF2* and *AoraSOD2*.

Primer	Sequence (5′-3′)
AoraOBP1-F	GCTGGTGTGAAGAAGGCTCT
AoraOBP1-R	GCTGGTGTGAAGAAGGCTCT
AoraOBP2-F	TATATTGGCGTCGGGGTGTG
AoraOBP2-R	TTCATCTTTCGGTGCTGCCT
AoraOBP3-F	TACAACAGGGCTCGGCTTTT
AoraOBP3-R	ATCTTCCCCGCCTTGTTTGT
AoraOBP4-F	GCTGGTAGCTTTCGCTTGTG
AoraOBP4-R	AGTGGTCCCGGTCTTACTGA
AoraOBP5-F	AAGACCATCCCGTGGAAGTG
AoraOBP5-R	CGTCGTACATTCCTTTGGCG
AoraOBP6-F	GCCTGCAGTTACAAAAGGGC
AoraOBP6-R	GCCTGCAGTTACAAAAGGGC
AoraOBP7-F	CCCTGCTTTTTGGCTTGCAT
AoraOBP7-R	CCCTGCTTTTTGGCTTGCAT
AoraOBP8-F	GCGGCTTTGTCAACGATTCA
AoraOBP8-R	TTGTTTCGCGCTTTGTCTGG
AoraOBP9-F	CCCTGCTTTTTGGCTTGCAT
AoraOBP9-R	GCGCAATGTTTCGCCAATTC
AoraOBP10-F	GCGCAATGTTTCGCCAATTC
AoraOBP10-R	GGTTCACAGAAGTGCAAGCC
AoraOBP11-F	TCACGGAAGATATGGTCGGC
AoraOBP11-R	GCATCCTTCATTTCTGGCGG
AoraOBP12-F	TCACGGAAGATATGGTCGGC
AoraOBP12-R	GCATCCTTCATTTCTGGCGG
AoraOBP13-F	TTGGCCGTGTAAAAGCAAGC
AoraOBP13-R	ACGCTTCGCACAAATCCTTG
AoraOBP14-F	CGAAGCGATGACGATGAAGC
AoraOBP14-R	ACGCCGTCTTTGATAGGGTC
AoraOBP15-F	GGTCGAGAAGACGCATGGAG
AoraOBP15-R	CCTTGTCGCCGTCTTTCACT
AoraOBP16-F	ATGTCTTACTTGCTGGCGCT
AoraOBP16-R	CCGACGCGATCTCTACCATC
AoraOBP17-F	ATGCGAGGCAGAAACTGGAG
AoraOBP17-R	CTCGTACGCGATGCTCTTCA
AoraOBP18-F	GAAATAGCGCGCATCGTCAC
AoraOBP18-R	CTGTCTCTTGTCTGCCGCTT
AoraOBP19-F	TATCCGCTGCAAGTCGACAG
AoraOBP19-R	TGCGTGCAGTCGTTGAAAAC
AoraOBP20-F	CCGGTACGATGGTGGACTTC
AoraOBP20-R	AAGCATTGTCCAGAGCGTGT
AoraOBP21-F	AGATCGGCGTAGTCAAGCAG
AoraOBP21-R	TCGCAGTTTTCGGAGACAGT
AoraOBP22-F	TGTGTGACCCTCGGTAAGGA
AoraOBP22-R	TGACGTTCTTCCCATCCTGC
AoraOBP23-F	ACAGTCACATGACGGACCAG

AoraOBP23-R	CGGCCTTGTAGTGGAAGCAT
AoraOBP24-F	CTGACCGACGAGCAGAAAGA
AoraOBP24-R	GCACGTACTTCTTCAACGGC
AoraOBP25-F	ATGCCGGAGCAGACCTAATG
AoraOBP25-R	TTTTCGCAGCCATGTCCTCT
AoraCSP1-F	CAGCTGGAGCAGACCCTG
AoraCSP1-R	CGTACTGGCGTACGATCCTC
AoraCSP2-F	AGACGCCCTCAACGATAAGC
AoraCSP2-R	ACGATCTTGGCCCACTCTTG
AoraCSP3-F	CGTCATCTACCTCACGAGCC
AoraCSP3-R	TTGACGTAGCCAGTCAGCAG
AoraCSP4-F	CAACGTCGACTTGGACCAGA
AoraCSP4-R	TTCAGGGTCGTACTTGTCGC
AoraCSP5-F	CGACAGCGTAGATTTGAGCG
AoraCSP5-R	CGTTGGGTCTCCGTACACTT
AoraCSP6-F	CTGGCCAACAAGAGACTGCT
AoraCSP6-R	GTCATGGCGTCGTTTACGTG
AoraCSP7-F	ACTGCATACTCGACAAGGGC
AoraCSP7-R	GCATTTCTCGCAACCCGTTT
AoraCSP8-F	ACACCAAGTGCTTCCTCGAC
AoraCSP8-R	ACTGCTTCTGCTTCGGAGTG
AoraCSP9-F	TAAGCAGAAGGGACTCGTGC
AoraCSP9-R	GTCGGGGTCGTATTTGTCCA
AoraCSP10-F	GTTTGTTGGCGTGTGCTGTA
AoraCSP10-R	CCTTCGTTTTGGCTTCGTCC
AoraCSP11-F	TGGCATCTATCGTGCTGGTC
AoraCSP11-R	CACAGGGTGTCCGATCTAGC
AoraCSP12-F	TCTCGTTGTCTGTGCCTTGG
AoraCSP12-R	TGGCGTCGAAATCGTAGTCC
AoraCSP13-F	TATCATCTGCTTGGGAGCGG
AoraCSP13-R	AATGCGCGCAATGGTCTTTC
AoraCSP14-F	TCATCTACTTGGGAGCGAGC
AoraCSP14-R	CCTCGCCAAGAATGCAGACA
AoraCSP15-F	CCGGAACAACCACAATGCAC
AoraCSP15-R	CAAGCATGCAGGCGATGTAG
AoraCSP16-F	GGTCGAGACCACCTGTGAAA
AoraCSP16-R	CCGGTAGGGTCGTTCTTCTT
AoraCSP17-F	ATCCTGTCAGTCCTCGTGGT
AoraCSP17-R	ATGCCATCGATGTCCAGGTC
AoraCSP18-F	GCTTACACCCAGTGTCTGCT
AoraCSP18-R	AGTTGCATTTGGCGCATGTT
AoraCSP19-F	TACGACAGCTTCGATGCCAA
AoraCSP19-R	CTGCTTCGGCGAGCATTTAG
AoraCSP21-F	AGGCCCTGGAAACTCATTGC
AoraCSP21-R	CGGGGTGCTTCTCCTTTAACT
AoraEF2-F	CCACCTACTCCGATGACAGC
AoraEF2-R	GAAGTTCTCTCCCCACAGCC
AoraSOD2-F	CAGCAAACATCACGCGACTT
AoraSOD2-R	AGTGGTTGATATGGCCACCG

Table S2. Quality control of RNA-seq data from various developmental stages of *A. orana*.

Sample	Raw reads	Raw bases	Clean reads	Clean bases	Error rate(%)	Q20(%)	Q30(%)	GC content(%)	rRNA Ratio(%)
Eg1	42362182	6396689482	42063262	6330017501	0.0119	99.22	96.04	46.42	0.431
Eg2	39374872	5945605672	39088166	5883540715	0.0119	99.22	96.05	47.05	0.553
Eg3	39723440	5998239440	39461338	5938966687	0.0118	99.25	96.12	47.27	0.429
L11	42568446	6427835346	42301706	6339585815	0.0118	99.23	96.15	49.08	0.642
L12	38830206	5863361106	38616674	5781821914	0.0119	99.21	96.04	50.22	1.019
L13	37491506	5661217406	37262146	5589883707	0.0119	99.22	95.97	48.56	1.344
L31	39344742	5941056042	39102482	5861588893	0.0118	99.24	96.12	49.61	1.147
L32	36646372	5533602172	36433780	5461885645	0.0119	99.24	96.09	49.61	1.543
L33	38666736	5838677136	38424620	5758666050	0.0119	99.22	95.99	48.97	1.488
L51	40781214	6157963314	40538450	6079648480	0.0118	99.27	96.17	49.34	1.776
L52	42766102	6457681402	42532590	6370144262	0.0118	99.27	96.21	49.31	2.727
L53	41400822	6251524122	41190972	6167977638	0.0119	99.24	96.07	50.32	3.861
PF1	43647230	6590731730	43380802	6529141844	0.0117	99.30	96.31	46.26	1.064
PF2	41349902	6243835202	41129152	6188471492	0.0118	99.29	96.23	47.99	0.630
PF3	45617458	6888236158	45320616	6822658729	0.0117	99.29	96.30	46.52	0.977
PM1	39293816	5933366216	39035128	5876326981	0.0119	99.25	96.08	48.50	1.156
PM2	39482852	5961910652	39236354	5906248140	0.0118	99.26	96.13	48.77	1.172
PM3	42952496	6485826896	42722172	6425207343	0.0117	99.30	96.32	47.63	0.920
AF1	39249826	5926723726	39043038	5874997251	0.0117	99.31	96.35	46.27	0.673
AF2	36734452	5546902252	36549818	5500167304	0.0117	99.32	96.37	46.37	1.068
AF3	37055782	5595423082	36862932	5548183975	0.0117	99.33	96.38	45.78	0.931
AM1	42586638	6430582338	42331724	6366053482	0.0118	99.28	96.28	46.45	1.780
AM2	43252138	6531072838	42979520	6462064398	0.0118	99.28	96.28	46.14	1.345
AM3	43467250	6563554750	43214752	6497277525	0.0118	99.27	96.19	46.83	1.156

Note: Eg, L1, L3, L5, PM, PF, AM, and AF represent whole bodies of eggs, 1st-instar larvae, 3rd-instar larvae, 5th-instar larvae, male pupae, female pupae, male adults, and female adults, respectively.

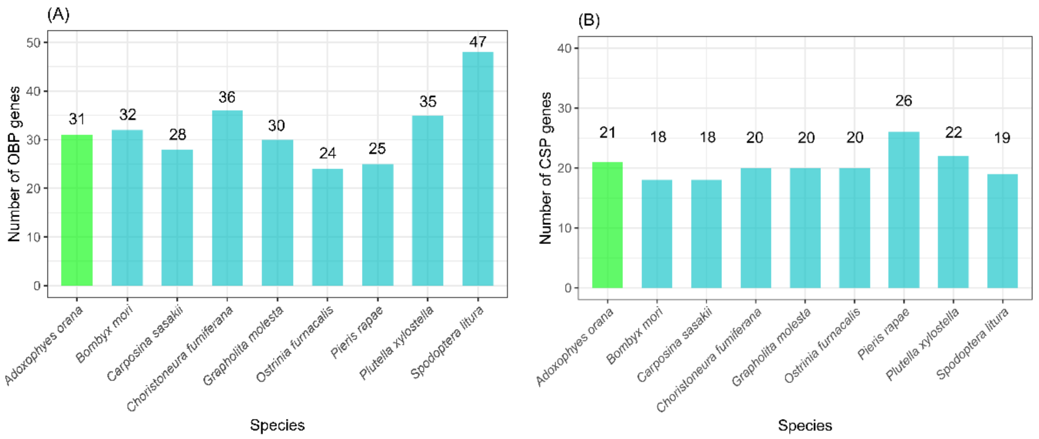

Figure S1. Gene number counts and phylogenetic analysis of OBPs across nine lepidopteran species. The number of (A) OBP and (B) CSP genes in *Adoxophyes orana* is symbolized by the green bar, while other species are represented by cyan bars.

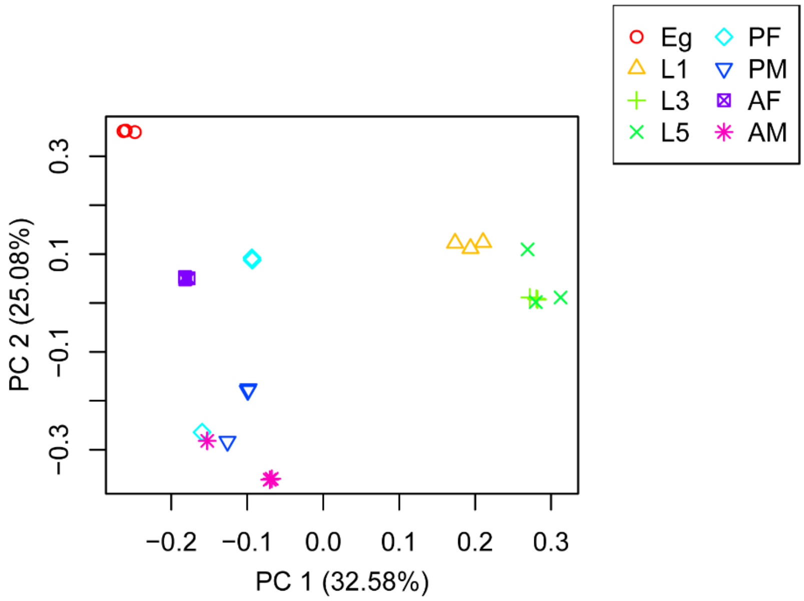

Figure S2. PCA analysis of RNA-seq data across various developmental stages of *A. orana*. Eg, L1, L3, L5, PM, PF, AM, and AF represent whole bodies of eggs, 1st-instar larvae, 3rd-instar larvae, 5th-instar larvae, male pupae, female pupae, male adults, and female adults, respectively.
